# The Receptor-like Kinase TaCRK-7A Inhibits *Fusarium pseudograminearum* Growth and Mediates Resistance to Fusarium Crown Rot in Wheat

**DOI:** 10.3390/biology10111122

**Published:** 2021-11-01

**Authors:** Tianci Wu, Feilong Guo, Gangbiao Xu, Jinfeng Yu, Li Zhang, Xuening Wei, Xiuliang Zhu, Zengyan Zhang

**Affiliations:** 1 Institute of Crop Sciences, The National Key Facility for Crop Gene Resources and Genetic Improvement, Chinese Academy of Agricultural Sciences, Beijing 100081, China; wtc19961110@163.com (T.W.); guofeilong1117@163.com (F.G.); weixuening@caas.cn (X.W.); 2 The Laboratory of Forestry Genetics, Central South University of Forestry and Technology, Changsha 410004, China; gangbiaoxu@163.com; 3 College of Plant Protection, Shandong Agricultural University, Tai’an 271018, China; jfyu@sdau.edu.cn (J.Y.); zli@sdau.edu.cn (L.Z.)

**Keywords:** antifungal activity, receptor-like kinase with DUF26 domain-containing, Fusarium crown rot resistance, *Fusarium pseudograminearum*, wheat (*Triticum aestivum*)

## Abstract

**Simple Summary:**

Fusarium crown rot (FCR), caused by a soil-borne fungus *Fusarium pseudograminearum*, is one of the most destructive diseases of cereal crops, including wheat (*Triticum aestivum*) in many countries. It is vital to isolate resistance genes for improving crop resistance. Herein, we report the positive function of the wheat DUF26 domain-containing receptor-like kinase TaCRK-7A in the host resistance response to the pathogen *F. pseudograminearum* attack. The purified TaCRK-7A protein directly inhibited *F. pseudograminearum* mycelial growth. The *TaCRK-7A* transcript was elevated upon *F. pseudograminearum* infection and the transcript induction was higher in resistant wheat genotypes than in susceptible wheat genotypes. Knocking down of *TaCRK-7A* compromised resistance of wheat to FCR and significantly reduced the transcript levels of defense genes in wheat. This study provides a novel insight into the wheat immune responses to *F. pseudograminearum*.

**Abstract:**

The fungus *F. pseudograminearum* can cause the destructive disease Fusarium crown rot (FCR) of wheat, an important staple crop. Functional roles of FCR resistance genes in wheat are largely unknown. In the current research, we characterized the antifungal activity and positive-regulatory function of the cysteine-rich repeat receptor-like kinase TaCRK-7A in the defense against *F. pseudograminearum* in wheat. Antifungal assays showed that the purified TaCRK-7A protein inhibited the growth of *F. pseudograminearum*. *TaCRK-7A* transcript abundance was elevated after *F. pseudograminearum* attack and was positively related to FCR-resistance levels of wheat cultivars. Intriguingly, knocking down of *TaCRK-7A* transcript increased susceptibility of wheat to FCR and decreased transcript levels of defense-marker genes in wheat. Furthermore, the transcript abundances of *TaCRK-7A* and its modulated-defense genes were responsive to exogenous jasmonate treatment. Taken together, these results suggest that TaCRK-7A can directly inhibit *F. pseudograminearum* growth and mediates FCR-resistance by promoting the expression of wheat defense genes in the jasmonate pathway. Thus, *TaCRK-7A* is a potential gene resource in FCR-resistant wheat breeding program.

## 1. Introduction

Fusarium crown rot (FCR) is a destructive disease of wheat (*Triticum aestivum* L.) and barley (*Hordeum vulgare* L.) worldwide [1]. It causes serious yield loss [2]. The soil-borne fungus *Fusarium pseudograminearum* is a major causal pathogen responsible for FCR disease [1,2,3]. Usage of resistant wheat cultivars is one effective way to control FCR in wheat. It is crucial to isolate the resistance genes for molecular breeding wheat with FCR resistance. Several groups already mapped wheat QTLs conferring resistance to FCR, including the QTLs on 3B, 6A, 4D, and 7A [1,4,5,6]. Most recently, a paper reported the wheat dirigent gene *TaDIR-B1* with negative role, whose loss-of-function enhances FCR-resistance in wheat [7]. However, functional roles of FCR resistance genes in wheat are largely unknown.

In Arabidopsis and crop plants, several cysteine-rich repeat receptor-like protein kinases (CRKs) have been shown to confer resistance or be involved in the plant innate immune responses to bacterial and fungal pathogens [8,9,10,11,12]. The protein sequences of CRKs all include an intracellular serine/threonine protein kinase domain, a transmembrane domain, and two copies of the extracellular Domain 26 of Unknown Function (DUF26, antifungal domain) each with a plant specific cysteine-rich motif [9,11]. The secreted ginkbilobin2 (Gnk2) protein from gymnosperm *Ginkgo biloba* comprises a single DUF26 domain and has been demonstrated to have antifungal activity [13]. The fungal mannose binding of three residues (asparagine-11, arginine-93, and glutamate-104) in its DUF26 domain has been determined to be necessary for the antifungal activity of Gnk2 [13,14]. In our previous study, a wheat CRK protein TaCRK3 was shown to defend against the wheat sharp eyespot pathogen *Rhizoctonia cerealis* [15]. The gene sequence is matched with the sequence TraesCS7A02G105100.1 and thus was named TaCRK-7A. However, no study about the functional roles of wheat CRK proteins in host resistance responses to *F. pseudograminearum* has been reported yet.

In this current report, we examined in vitro inhibition activity of the purified TaCRK-7A protein against the growth of *F. pseudograminearum* and investigated its functional role in the wheat resistance to FCR caused by *F. pseudograminearum* infection. 

## 2. Materials and Methods

### 2.1. Materials and Treatments

Six wheat cultivars, including FCR-resistant cultivars (Nivat14, CI12633, Chinese Spring), and susceptible cultivars (Jimai 22, Yangmai 6, and Yangmai 158) [16,17], were used to examine *TaCRK-7A* transcript profiles. 

The pathogenic fungus *F. pseudograminearum* strain WHF220 was isolated from the FCR-symptomatic wheat sheaths in Shandong by Prof. Jinfeng Yu and Dr. Li Zhang (Shandong Agricultural University, Tai’an, China).

To investigate the transcript profile and defensive role of *TaCRK-7A*, wheat plants at the early-tiller stage were inoculated with toothpicks harboring the well-developed mycelia of *F. pseudograminearum*. Furthermore, seedlings of wheat cultivar CI12633 at three-leaf stage were treated with 0.05 mM methyl jasmonate (MeJA, a jasmonate analog) and sampled after spraying for 0.5, 1, 3, 6, 12, and 24 h as described by Zhang et al. [18]. 

### 2.2. Assay on Inhibition of Purified TaCRK-7A against F. pseudograminearum Mycelial Growth 

According to the protocol described previously [15], the His-TF-TaCRK-7A recombinant or His-TF proteins were separately expressed in *Escherichia coli* DE3 cells, secreted into the culture supernatant, and purified. The purified His-TF-TaCRK-7A or His-TF control proteins were separately added into the middle pore of the potato dextrose agar (PDA) medium plate. Subsequently, the liquid mycelia of *F. pseudograminearum* was inoculated into the each pore and subjected to further incubation at 25 °C for 5 days [15]. Photographs for *F. pseudograminearum* mycelia/hyphae growth status were taken on the fifth day. These assays were repeated three times.

### 2.3. Assesment on Defense Role of TaCRK-7A in Wheat against FCR

By means of the barley yellow dwarf virus (BSMV)-based virus-induced gene silencing (VIGS) experiments as described [19], the BSMV:TaCRK-7A VIGS construct was prepared [15] and the *TaCRK-7A* transcript in the resistant wheat cultivar (cv.) CI12633 plants was knocked down. Twenty days after the virus transfection, *TaCRK*-7A-silenced or the BSMV:GFP-infected (control) wheat plants were further inoculated with *F. pseudograminearum* strain WHF220 as previously described [7]. At ~28 d post inoculation (dpi) with the fungal pathogen, the average lesion length and width of FCR on the inoculated sheaths of these plants were measured, and the infection types of these wheat plants were rated [1,17]. In addition, Trypan blue staining for *F. pseudograminearum* hyphae was used to assess the defense role of *TaCRK-7A* following Zhang et al. 2012 [18]. The experiments were performed two batches. At least 15 plants for each construct were tested in each batch. 

### 2.4. RNA Extraction and Real-Time Quantitative PCR (qRT-PCR) Analysis

The extraction of RNAs from wheat samples was conducted by Trizol reagent (Invitrogen, Carlsbad, CA, USA). Then, the RNA was purified and was reverse-transcribed into cDNA by using the FastQuant RT Kit (Tiangen, Beijing, China). The transcript levels of *TaCRK-7A* and four defense-marker genes in wheat plants were tested by using the gene-specific primers [15] and the qRT-PCR technique [15,18,19]. The qRT-PCR was conducted by using SYBR Premix Ex Taq kit (TaKaRa, Otsu, Japan) on an ABI 7500 instrument (Applied Biosystems, Waltham, MA, USA). The relative transcript levels of the tested genes were calculated with the 2^−ΔΔCT^ method [20]. 

## 3. Results and Discussion

### 3.1. The Purified TaCRK-7A Protein Directly Inhibits F. pseudograminearum Hyphae Growth

**Figure 1:**
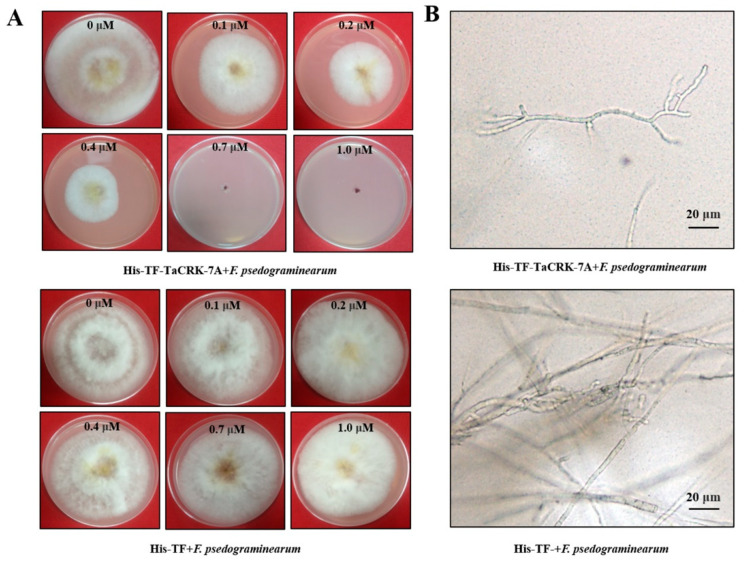
Assay on inhibition of the purified TaCRK-7A protein against *F. pseudograminearum* hyphae growth. (**A**) The growth status of liquid *F. pseudograminearum* mycelia on the PDA medium plus His-TF-TaCRK-7A protein or plus His-TF (control) at different concentration (0, 0.1, 0.2, 0.4, 0.7, and 1 μM) on the fifth day. (**B**) Light microscopic photos on the hyphae of *F. pseudograminearum* after being treated by His-TF-TaCRK-7A or His-TF (control). Scale bars = 20 μm.

The deduced TaCRK-7A protein sequence includes an intracellular serine/threonine protein kinase domain, a transmembrane domain, and two DUF26 domains with the three conversed amino acids (asparagine, arginine, and glutamate) that are required for mannose binding activity and for antifungal activity [13]. We performed in vitro antifungal assay as described by Guo et al. 2021 [15]. The antifungal assay results showed that compared to the His-TF tag protein from the original vector pCOLD, the purified His-TF-TaCRK-7A protein could obviously inhibit the mycelial/hyphae growth of *F. pseudograminearum* strain WHF220 (Figure 1A,B). These data suggested that TaCRK-7A protein possesses antifungal activity against *F. pseudograminearum*. The activity is possibly due to the three conversed amino acids (asparagine, arginine, and glutamate) in the extracellular DUF26 domain. In our previous study, the purified DUF26 domain in TaCRK-7A, like the whole protein, could inhibit the mycelial/hyphae growth of *R. cerealis*, indicating that the DUF26 domain is required for the antifungal activity of TaCRK-7A protein [15]. Similarly, previous papers reported that the DUF26 domain-containing peptide Gnk2 in *G. biloba,* due to binding with mannose of fungal wall cells, could inhibit the growth of several plant-pathogenic fungi including *Fusarium* species [13,14]. Meanwhile, the maize DUF26-domain-containing peptide ZmAFP1 binds fungal mannose and displays antifungal activity only against the repetitive effector-coding gene *rsp3* mutant of the biotrophic fungus *Ustilago maydis*, but not against the fungal strain constitutively expressing *rsp3* [21].

### 3.2. TaCRK-7A Transcript Abundance Is Related to FCR-Resistance Degree of Wheat Accessions

**Figure 2:**
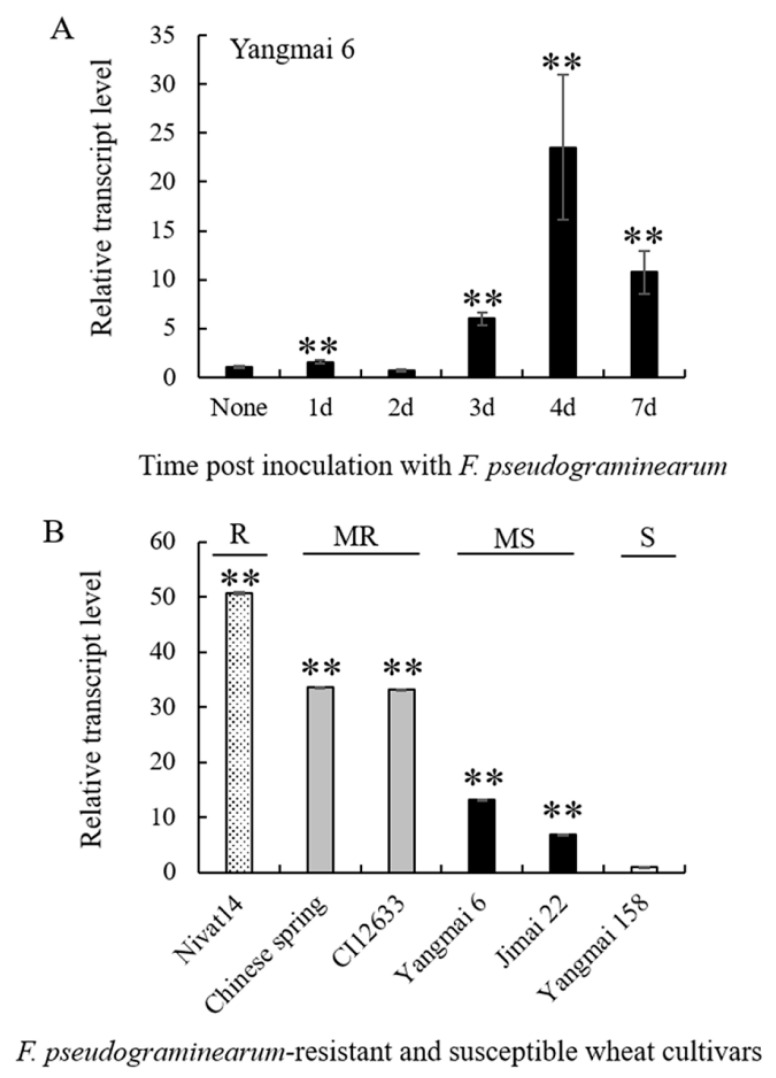
Transcript profiles of *TaCRK-7A* in the wheat response to *F. pseudograminearum* infection via qRT-PCR analysis. (**A**) *TaCRK-7A* transcript profile in the wheat cultivar Yangmai 6 levels at non-inoculation (none), 1, 2, 3, 4, and 7 d post inoculation with *F. pseudograminearum* strain WHF220. *TaCRK-7A* transcript level at none is set to 1. (**B**) Transcript patterns of *TaCRK-7A* in six wheat cultivars at 4 dpi with *F. pseudograminearum* WHF220. The gene transcript in the highly susceptible wheat cultivar Yangmai 158 was set to 1. R represents resistant wheat cv.; MR represents moderately resistant wheat cultivars; MS represents moderately susceptible wheat cultivars; S represents highly susceptible wheat cv.. *TaActin* was used as the internal control for gene expression. ** *p* < 0.01 (Student’s *t*-test) represents significant differences derived from three repeats. Bars indicate the standard deviation of the mean.

*TaCRK-7A* promoter sequence includes two TC-rich *cis*-acting elements that are reported to be involved in defense and stress responses [15]. Our qRT-PCR analyses showed that in comparison with non-inoculation, *TaCRK-7A* transcript abundance was significantly elevated in wheat after *F. pseudograminearum* attack and reached a peak at 4 dpi with the pathogen (Figure 2A), consistent with the promoter sequence characteristic. Intriguingly, the gene expression level at 4 dpi was related to the FCR-resistance degree of the wheat cultivars tested (Figure 2B). These data imply that *TaCRK-7A* might be involved in wheat resistance against FCR. To further explore the cause of the transcript difference, we cloned and sequenced the *TaCRK-7A* sequences from these six wheat cultivars. As a result, there was no genetic variation within the promoter and most gene-body sequences, but a single nucleotide polymorphism (SNP) existed in the third intron of the gene. Namely, a nucleotide “A” at position 1686-bp in two resistant wheat cultivars (Chinese spring and CI12633) was replaced by “G” at the site in the susceptible varieties Yangmai6, Jimai22, and Yangmai 158, as well as in the resistant cultivar Nivat 14 (Figure S1). Therefore, we supposed that the TaCRK-7A transcript difference between resistant and susceptible wheat cultivars might be associated with epigenetic events. Similarly, epigenetic events contributed to the transcription of *TaCYP81D5* conferring salinity tolerance, whereas no genetic variation within the promoter and the genebody sequences of *TaCYP81D5* appeared between tolerant and intolerant wheat cultivars [22]. 

Further sequence search indicated that in wheat genome, there is a paralogous gene *TaCRK-7D* with sequence identity number TraesCS7D02G099300.1. The TaCRK-7D protein also contains two DUF26 domains, and has 82.58% identity in amino aide sequence of TaCRK-7A protein. *TaCRK-7D* gene shares 89.70% identity with *TaCRK-7A* in nucleotide sequence. In fact, the qRT-PCR experiment showed that the transcript of *TaCRK-7D* was also induced by *F. pseudograminearum* infection (Figure S2). It is very interesting to investigate the effect of *TaCRK-7D* in the wheat resistance response to FCR in future.

### 3.3. TaCRK-7A Is Required for Resistance of Wheat to FCR

**Figure 3:**
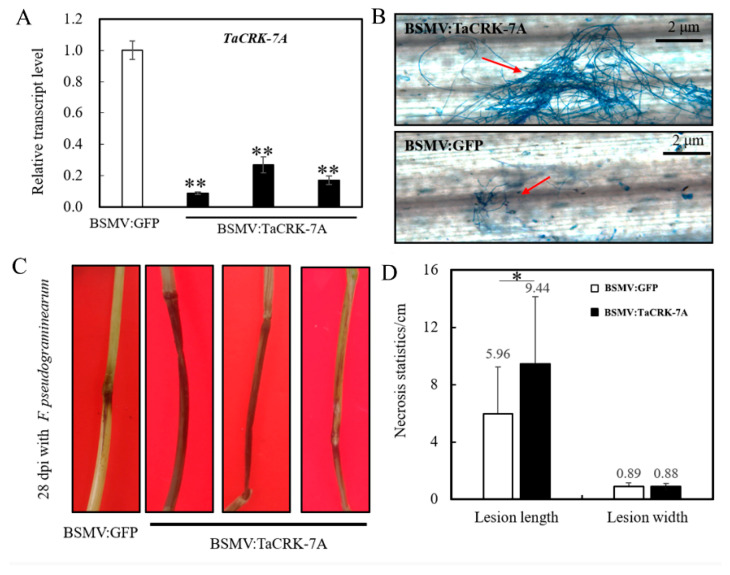
Knocking-down of *TaCRK-7A* increases susceptibility of the wheat cv. CI12633 to *F. pseudograminearum*. (**A**) *TaCRK-7A* transcript was markedly knocked down in BSMV:TaCRK7A-infected wheat CI12633 plants relative to BSMV:GFP-transfected CI12633 (control) plants at 12 d after the virus transfection. The transcript level in BSMV:GFP-transfected (control) plants was set to 1. *TaActin* was used as internal control. (**B**) Microscopic observation of the *F. pseudograminearum* hyphae on the fungus-infected sheaths of the BSMV:TaCRK-7A-silenced and BSMV:GFP-infected CI12633 plants. (**C**) FCR symptoms on the BSMV:GFP (control) and TaCRK-7A-silenced CI12633 sheaths at 28 dpi with *F. pseudograminearum* WHF220. (**D**) The average lesion length and width of the BSMV:GFP-transfected and BSMV:TaCRK-7A-silenced plants. ** *p* < 0.01 and * *p* < 0.5 (Student’s *t*-test) represent statistically significant differences derived from three repeats. Bars represent standard error of the mean.

To assess the function of *TaCRK*-*7A* in wheat defense against FCR caused by infection of *F. pseudograminearum*, we performed two batches of the functional assays in the mildly resistant wheat cv. CI12633 by means of VIGS plus disease evaluation. qRT-PCR assay showed that, at the virus transfection for 12 d, the *TaCRK-7A* transcript level was markedly decreased in BSMV:TaCRK-7A-transfected CI12633 plants compared to BSMV:GFP-infectedCI12633 (control) plants (Figure 3A). These *TaCRK-7A*-silenced and control plants were further inoculated with *F. pseudograminearum* strain WHF220. At 7 dpi with the fungal pathogen, microscopic observation indicated that more *F. pseudograminearum* hyphae appeared on the fungus-infected sheaths of BSMV:TaCRK-7A-silenced wheat plants than on those of BSMV:GFP-infected plants (Figure 3B). The FCR disease assessments showed that in comparison with BSMV:GFP-infected plants, *TaCRK*-*7A*-silenced plants displayed more susceptibility to FCR caused by *F. pseudograminearum* infection, such as necrosis length and disease severity (Figure 3C,D). In two batch experiments, the average infection types (ITs) on stems of *TaCRK-7A*-silenced plants were 4.09 ± 1.00 and 4.70 ± 0.71, whereas those for BSMV:GFP-transfected plants were 2.20 ± 0.87 and 3.85 ± 0.96. These data pointed to the conclusion that the functional TaCRK-7A, acting as a positive regulator, was required for wheat resistance to FCR caused by infection of *F. pseudograminearum*. The above results indicated *TaCRK-7A* transcript was induced by *F. pseudograminearum* from 1 to 7 d, and the transcripts in six wheat cultivars at 4 dpi were correlated with the resistance levels of the tested cultivars (Figure 2). Based on the previous experiences [7,15,19], it was deduced that the *TaCRK-7A* transcript at 7 dpi might be also related with the disease resistance. To ensure enough development of FCR disease in the VIGS plus the fungal pathogen experiments, we sampled these wheat sheaths inoculated with the fungus at 7 d for further hyphae staining and qRT-PCR analysis.

### 3.4. TaCRK-7A and Its Modulated-Defense Genes Were Responsive to JA Stimulus

**Figure 4:**
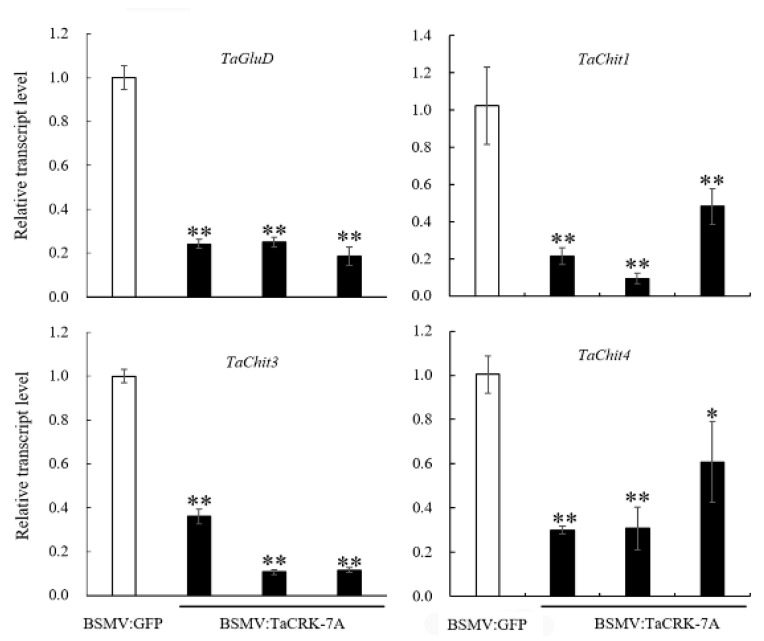
Silencing of *TaCRK-7A* reduced transcript abundances of defense genes in wheat CI12633 plants at 7 dpi with *F. pseudograminearum* WHF220. Relative transcript abundances of these defense genes in BSMV:TaCRK-7A-silenced CI12633 plants were quantified relative to those in BSMV:GFP-infected control plants. *TaActin* was used as internal control. Statistically significant differences were determined based on three replications using Student’s *t*-test (* *p* < 0.05, ** *p* < 0.01).

To explore the molecular mechanism underlying the FCR-resistance function of TaCRK-7A, we first examined if *TaCRK*-*7A* modulates the expression of wheat defense genes. These experiments showed that knocking-down of *TaCRK*-*7A* significantly decreased the transcript levels of four defense-marker genes in *TaCRK*-*7A*-silenced wheat plants at 7 dpi with *F. pseudograminearum* WHF220 (Figure 4). The tested wheat defense-marker genes include *PR2 (TaGluD)*, *chitinase 1* (*TaChit1*)*, chitinase 3* (*TaChit3*)*,* and *chitinase 4* (*TaChit4*). The data suggested that the functional TaCRK-7A might be upstream of these defense-marker genes and also up-regulated their expression levels during wheat resistance responses against *F. pseudograminearum* infection. The results support previous reports about CRKs acting as upstream regulatory factors [10,15].

**Figure 5:**
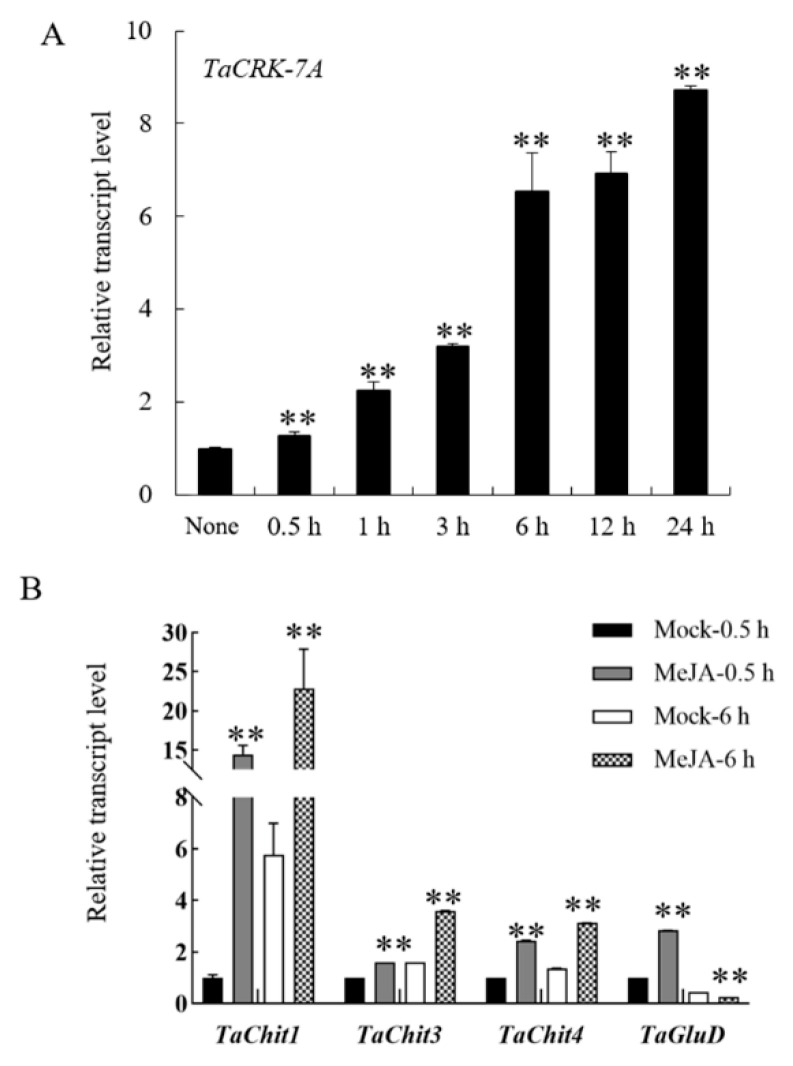
Transcript patterns of *TaCRK-7A* and its modulated-defense genes in wheat leaves treated with exogenous MeJA (0.05 mM). (**A**) Transcript profile of *TaCR-7A* in the wheat cv. CI12633 leaves treated by exogenous MeJA. The transcript level of *TaCRK-7A* in untreated wheat plants (none) is set to 1. (**B**) Transcript profiles of *TaChit1*, *TaChit3*, *TaChit4*, and *TaGluD* in wheat CI12633 leaves treated with exogenous MeJA. The transcription level of the tested gene in mock-treated (0.5 h) wheat plants is set to 1. Statistically significant differences (** *p* < 0.01) were analyzed based on three replications using Student’s *t*-test. Bars indicated standard error of the mean.

Furthermore, *TaCRK*-*7A* promoter contains jasmonate (JA)-responsive *cis*-acting elements (CGTCA-motif and TGACG-motif). Thus, we analyzed the transcript profile of *TaCRK*-*7A* in the wheat cv. CI12633 plants treated by external JA for 0.5, 1, 3, 6, 12, and 24 h. The analysis showed that compared to non-treatment (none), *TaCRK*-*7A* transcript level was significantly increased after JA treatment from 0.5 h to 24 h, and the induction reached the first peak at 6 h (Figure 5A). Moreover, the promoter sequences of *TaChit1, TaChit3*, *TaChit4,* and *TaGluD,* whose transcripts were positively modulated by TaCRK-7A (Figure 4), all contain JA-responsive *cis*-acting elements. Thus, transcript profiles of these defense-marker genes were tested in the wheat cv. CI12633 plants treated with JA for 0.5 h and 6 h. As a result, compared with the mock treatment, transcript levels of *TaChit1*, *TaChit3*, *TaChit4,* and *TaGluD* were significantly increased after JA treatment (Figure 5B). These results suggest that *TaCRK-7A* and its modulated-defense genes (*TaChit1*, *TaChit3*, *TaChit4,* and *TaGluD*) were all responsive to the JA stimulus. Similarly, a previous paper reported that *GbCRK18,* a cotton CRK-encoding gene, was also responsive to exogenous JA stimulus [23]. Taken together, these results suggest that TaCRK-7A mediates wheat resistance to FCR through promoting the expression of four wheat defense-marker genes in JA signaling. 

## 4. Conclusions

The current research revealed a novel defense role of the wheat RLK TaCRK-7A. TaCRK-7A not only directly inhibits the hyphae growth of *F. pseudograminearum*, but also mediates wheat resistance to FCR through promoting the expression of wheat defense-marker genes in JA signaling pathway. This study sheds a light on the molecular mechanisms underlying wheat defense against *F. pseudograminearum* and deepens understanding of the mechanisms of CRKs in plant–fungi interactions. Practically, *TaCRK-7A* is a promising gene for molecular breeding wheat with resistance to FCR caused by *F. pseudograminearum*.

## Data Availability

Not applicable.

## Supplementary Materials

The following are available online at https://www.mdpi.com/article/10.3390/biology10111122/s1. Figure S1: Sequence alignment among sequences in the third intron of TaCRK-7A among these six wheat cultivars. The blue word represents the SNP site. Figure S2: *TaCRK-7D* transcript profile in the wheat cv. Yangmai 6 leaves inoculated with *F. pseudograminearum* strain WHF220 for 1, 2, 3, 4, and 7 d or non-treatment (none). The transcript level at none is set to 1. *TaActin* was used as the internal control for gene expression. ** *p* < 0.01 (Student’s *t*-test) represents significant differences derived from three repeats. Bars indicate the standard deviation of the mean.

## References

[1] Yang X., Pan Y., Singh P.K., He X., Ren Y., Zhao L., Zhang N., Cheng S., Chen F. (2019). Investigation and genome-wide association study for Fusarium crown rot resistance in Chinese common wheat. BMC Plant. Biol..

[2] Smiley R.W., Gourlie J.A., Easley S.A., Patterson L.M., Whittaker R.G. (2005). Crop Damage Estimates for Crown Rot of Wheat and Barley in the Pacific Northwest. Plant. Dis..

[3] Kazan K., Gardiner D.M. (2018). Fusarium crown rot caused by Fusarium pseudograminearum in cereal crops: Recent progress and future prospects. Mol. Plant. Pathol..

[4] Ma J., Li H.B., Zhang C.Y., Yang X.M., Liu Y.X., Yan G.J., Liu C.J. (2010). Identification and validation of a major QTL conferring crown rot resistance in hexaploid wheat. Theor. Appl. Genet..

[5] Zheng Z., Ma J., Stiller J., Zhao Q., Feng Q., Choulet F., Feuillet C., Zheng Y.L., Wei Y., Han B. (2015). Fine mapping of a large-effect QTL conferring Fusarium crown rot resistance on the long arm of chromosome 3B in hexaploid wheat. BMC Genom..

[6] Poole G.J., Smiley R.W., Paulitz T.C., Walker C.A., Carter A.H., See D.R., Garland-Campbell K. (2012). Identification of quantitative trait loci (QTL) for resistance to Fusarium crown rot (Fusarium pseudograminearum) in multiple assay environments in the Pacific Northwestern US. Theor. Appl. Genet..

[7] Yang X., Zhong S., Zhang Q., Ren Y., Sun C., Chen F. (2021). A loss-of-function of the dirigent gene TaDIR-B1 improves resistance to Fusarium crown rot in wheat. Plant. Biotechnol. J..

[8] Saintenac C., Cambon F., Aouini L., Verstappen E., Ghaffary S.M.T., Poucet T., Marande W., Berges H., Xu S., Jaouannet M. (2021). A wheat cysteine-rich receptor-like kinase confers broad-spectrum resistance against Septoria tritici blotch. Nat. Commun..

[9] Chen K., Fan B., Du L., Chen Z. (2004). Activation of hypersensitive cell death by pathogen-induced receptor-like protein kinases from Arabidopsis. Plant. Mol. Biol..

[10] Acharya B.R., Raina S., Maqbool S.B., Jagadeeswaran G., Mosher S.L., Appel H.M., Schultz J.C., Klessig D.F., Raina R. (2007). Overexpression of CRK13, an Arabidopsis cysteine-rich receptor-like kinase, results in enhanced resistance to Pseudomonas syringae. Plant. J..

[11] Yadeta K.A., Elmore J.M., Creer A.Y., Feng B., Franco J.Y., Rufian J.S., He P., Phinney B., Coaker G. (2017). A Cysteine-Rich Protein Kinase Associates with a Membrane Immune Complex and the Cysteine Residues Are Required for Cell Death. Plant. Physiol..

[12] Kimura S., Hunter K., Vaahtera L., Tran H.C., Citterico M., Vaattovaara A., Rokka A., Stolze S.C., Harzen A., Meißner L. (2020). CRK2 and C-terminal Phosphorylation of NADPH Oxidase RBOHD Regulate Reactive Oxygen Species Production in Arabidopsis. Plant. Cell.

[13] Miyakawa T., Hatano K., Miyauchi Y., Suwa Y., Sawano Y., Tanokura M. (2014). A secreted protein with plant-specific cysteine-rich motif functions as a mannose-binding lectin that exhibits antifungal activity. Plant. Physiol..

[14] Sawano Y., Miyakawa T., Yamazaki H., Tanokura M., Hatano K. (2007). Purification, characterization, and molecular gene cloning of an antifungal protein from Ginkgo biloba seeds. Biol. Chem..

[15] Guo F., Wu T., Shen F., Xu G., Qi H., Zhang Z. (2021). The cysteine-rich receptor-like kinase TaCRK3 contributes to defense against Rhizoctonia cerealis in wheat. J. Exp. Bot..

[16] Zhou M.P., Yao J.B., Zhang G.H., Yu G.H., Ma H.X. (2016). Screening of germplasm and establishment of new evaluation method for the resistance to wheat crown rot. J. Plant. Genet. Resour..

[17] Zhang P., Huo Y., Zhou M.P., Yao J.B., Ma H.X. (2009). Identification and evaluation of wheat germplasm resistance to crown rot caused by Fusarium graminearum. J. Plant. Genet. Resour..

[18] Zhang Z., Liu X., Wang X., Zhou M., Zhou X., Ye X., Wei X. (2012). An R2R3 MYB transcription factor in wheat, TaPIMP1, mediates host resistance to Bipolaris sorokiniana and drought stresses through regulation of defense- and stress-related genes. New Phytol..

[19] Zheng H., Dong L., Han X., Jin H., Yin C., Han Y., Li B., Qin H., Zhang J., Shen Q. (2020). The TuMYB46L-TuACO3 module regulates ethylene biosynthesis in einkorn wheat defense to powdery mildew. New Phytol..

[20] Livak K.J., Schmittgen T.D. (2001). Analysis of relative gene expression data using real-time quantitative PCR and the 2(-Delta Delta C(T)) Method. Methods.

[21] Ma L.S., Wang L., Trippel C., Mendoza-Mendoza A., Ullmann S., Moretti M., Carsten A., Kahnt J., Reissmann S., Zechmann B. (2018). The Ustilago maydis repetitive effector Rsp3 blocks the antifungal activity of mannose-binding maize proteins. Nat. Commun..

[22] Wang M., Yuan J., Qin L., Shi W., Xia G., Liu S. (2020). TaCYP81D5, one member in a wheat cytochrome P450 gene cluster, confers salinity tolerance via reactive oxygen species scavenging. Plant. Biotechnol. J..

[23] Li T.G., Zhang D.D., Zhou L., Kong Z.Q., Hussaini A.S., Wang D., Li J.J., Short D.P.G., Dhar N., Klosterman S.J. (2018). Genome-Wide Identification and Functional Analyses of the CRK Gene Family in Cotton Reveals GbCRK18 Confers Verticillium Wilt Resistance in Gossypium barbadense. Front. Plant. Sci..

