# Supplementary figures and images for: The Receptor-like Kinase TaCRK-7A Inhibits Fusarium pseudograminearum Growth and Mediates Resistance to Fusarium Crown Rot in Wheat
Source: Biology (Basel). 2021 Nov 1;10(11):1122. doi: 10.3390/biology10111122 (PMC8614996; doi:10.3390/biology10111122)

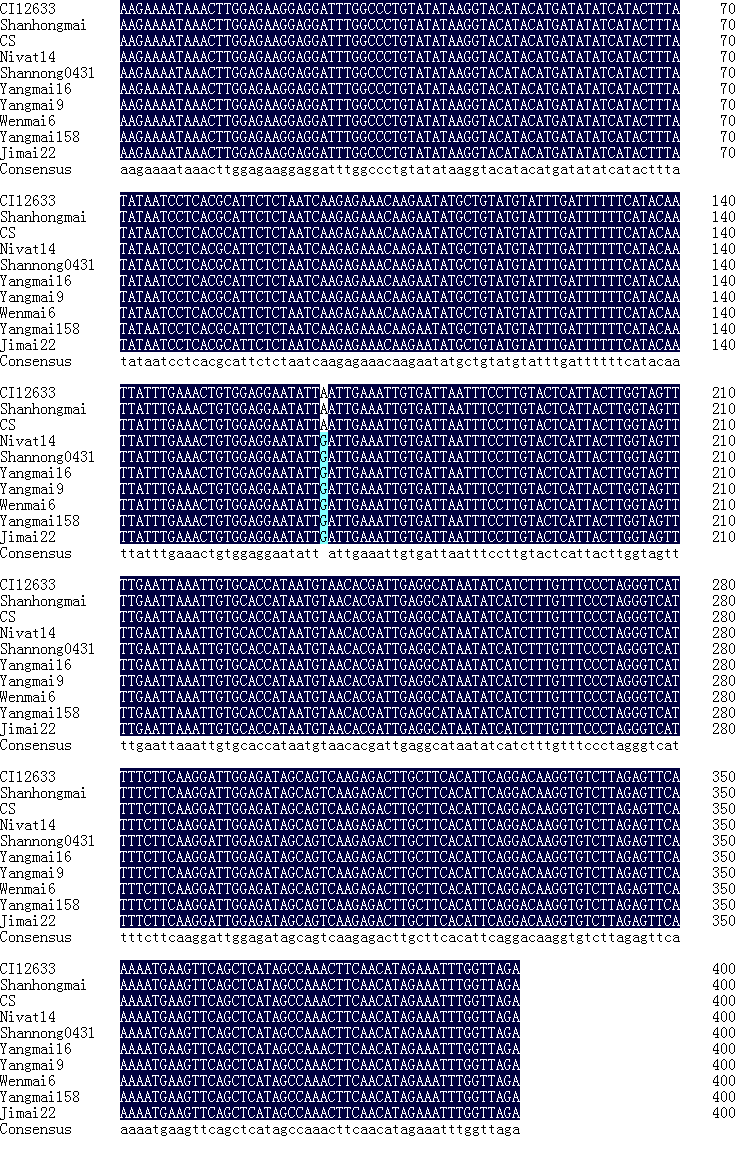

Supplement: Supplementary file 1 [file biology-10-01122-s001.zip › Figure S1.tif]

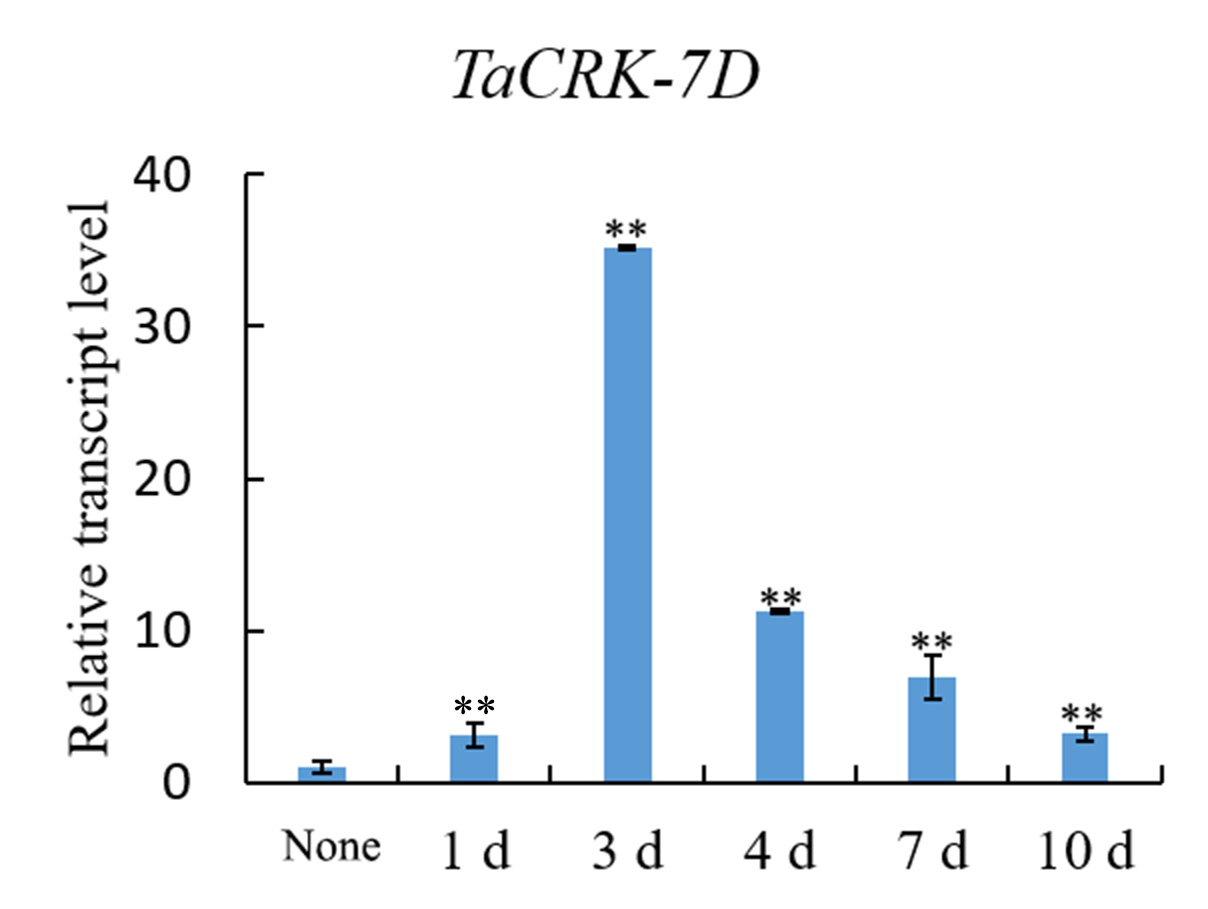

Supplement: Supplementary file 1 [file biology-10-01122-s001.zip › Figure S2.tif]
